# Prospective Cohort Study of Central Adiposity and Risk of Death in Middle Aged and Elderly Chinese

**DOI:** 10.1371/journal.pone.0138429

**Published:** 2015-09-16

**Authors:** Shaneda Warren Andersen, Xiao-Ou Shu, Yu-Tang Gao, Xianglan Zhang, Hui Cai, Gong Yang, Hong-Lan Li, Yong-Bing Xiang, Wei Zheng

**Affiliations:** 1 Division of Epidemiology, Department of Medicine, Vanderbilt Epidemiology Center, Vanderbilt-Ingram Cancer Center, Vanderbilt University School of Medicine, Nashville, Tennessee, United States of America; 2 Department of Epidemiology, Shanghai Cancer Institute, Renji Hospital, Shanghai Jiaotong University School of Medicine, Shanghai, China; University of Chieti, ITALY

## Abstract

Asians have high prevalence of central obesity despite the low prevalence of general obesity. We evaluated associations between the central obesity measure, waist-hip ratio (WHR) with total and cause-specific mortality in middle-aged and elderly Chinese participants. Data arise from two prospective population-based cohort studies: the Shanghai Men’s Health Study involves 53,425 men (participation rate = 74.0%), age 40–74 at baseline, and the Shanghai Women’s Health Study involves 63,017 women (participation rate = 92.7%), age 40–70 at baseline. Information on lifestyle factors and anthropometric measurements were taken at baseline interview. Vital status and causes of death were obtained via surveys and annual linkages to relevant Shanghai registries through December 31, 2011. After median follow-up time of 7.5 years for the Shanghai Men’s Health Study and 13.2 years for the Shanghai Women’s Health Study, there were 2,058 and 3,167 deaths, respectively. In models adjusted for BMI and other potential confounders, WHR was associated with all-cause mortality; hazard ratios (HRs) (95% confidence intervals) across the first to fifth quintile increased from 1 (Reference), 1.10 (0.95,1.27), 1.21 (1.04,1.41), 1.11 (0.96,1.30), to 1.42 (1.22,1.65) in men and from 1 (Reference), 1.10 (0.96,1.27), 1.11 (0.97,1.27), 1.20 (1.05,1.37), to 1.48 (1.30,1.69) in women. WHR had a stronger association with cardiovascular disease, with multivariate-adjusted HRs of 1.5 to 1.7 observed for the highest versus lowest quintile of WHR. Dose-response associations were also seen for cancer and other-cause deaths. Stratified analyses suggested a stronger association with mortality among normal weight (BMI <25) than over-weight (BMI ≥25) individuals. Positive associations with mortality were observed in subgroups defined by follow-up duration, comorbidity, age, smoking, and physical activity. Greater central adiposity is associated with increased mortality in Chinese adults, even among individuals with low BMI. Physicians and the public should be aware of central adiposity’s independent effects on health.

## Introduction

Greater adiposity is associated with multiple adverse health conditions including diabetes, coronary heart disease, and several cancer types [1]. Most epidemiologic studies suggest an overall J-shaped association between body mass index (BMI) and all-cause mortality, with the lowest risk typically observed among those with normal weight BMI as defined by the World Health Organization [2–5]. The majority of these studies have been conducted in middle aged white American and European samples. In a recent pooled analysis including more than one million Asian participants, we found the same BMI range associated with the lowest all-cause mortality risk to that observed in studies conducted using participants of European ancestry [6], however the magnitude of the increased risk associated with high BMI was more modest in Asians. Conversely, Asian populations tend to experience adverse health outcomes, such as diabetes, at lower BMI than the traditional overweight and obese cutoffs defined mostly using data obtained from populations of European ancestry [7–9]. Some studies show that for the same BMI, Asians may have more body fat than North American whites or Europeans [7,10,11]. Therefore, the use of BMI alone to infer health risks in Asians may underestimate excess adiposity’s detrimental health effects.

Considering measures of central fat distribution in addition to BMI may better quantify disease and mortality risk. Most studies conducted in North American and European samples report an approximately 50% increased mortality risk in individuals with the largest compared to the smallest central adiposity measures [2,12–17]. Few studies have evaluated central adiposity in association with mortality in Asians who account for approximately 60% of the world population. In a recent systematic review of published data regarding the association of general and central obesity in relation to mortality, no Asian data were included because of limited published studies for adequate evaluation. To quantify the association of central adiposity with mortality outcomes in Asians, we analyzed data from two large prospective cohort studies conducted among Chinese men and women in Shanghai. The majority of men participants smoke, whereas few women smoke, providing a unique setting to evaluate whether the relationship between waist-hip ratio and mortality is similar across smoking habits. Furthermore, because several previous studies suggested that the association between central adiposity and mortality may be stronger in individuals who have low BMI [2], are less physically active [15], and are former or current smokers [2], we sought to investigate these potential modifying associations.

## Materials and Methods

### Study population

The designs and methods of the population-based prospective Shanghai Women’s Health Study and Shanghai Men’s Health Study have been previously described in detail [18–20]. In brief, the Shanghai Women’s Health Study recruited 74942 women aged 40 to 70 years from 1996–2000 (participation rate = 92.7%); the Shanghai Men’s Health Study enrolled 61482 men aged 40 to 74 years from 2002–2006 (participation rate = 74.0%). The Vanderbilt University Medical Center and Shanghai Cancer Institute institutional review boards for human research in the United States and China approved the studies and the informed consent procedure. All participants provided written consent to participate in the study.

### Data collection

Structured questionnaires were used by trained interviewers at baseline to obtain information on demographics, lifestyle, and medical history. Anthropometric measurements were taken by interviewers according to standard protocol. Participants were asked to wear light indoor clothing when measured for weight, height, and circumferences of the waist and hips. Weight was measured to the nearest 0.1 kg using a digital scale that was calibrated every six months. Waist circumference was measured at 2.5 cm above the umbilicus and hip circumference at the level of maximum width of the buttocks with the subject in a standing position. All measurements were taken twice. A third measurement was taken if the difference of the first two measurements was greater than 1 kg for weight or 1 cm for height and circumference measurements. Waist-hip ratio and BMI were calculated using the average of the two closest measurements.

### Outcome ascertainment

Information on vital status and cause of death was obtained by annual record linkages to the Shanghai Cancer Registry and the Shanghai Vital Statistics Registry and by in-person follow-up surveys conducted every two to three years. The present analysis includes outcome data through December 31, 2011. Mortality follow-up for these cohorts is virtually complete (>99%). For each death, the underlying cause of death was determined on the basis of death certificates and coded according to the *International Classification of Diseases*, *Ninth Revision*. The primary endpoints for analyses were deaths due to all-causes, cardiovascular disease (CVD) (codes 390–459), cancer (codes 140–208), and all other-causes.

### Statistical analysis

Participants excluded from analysis were: missing waist or hip measurements (68 men, 25 women), missing covariate data (173 men, 68 women), lost to follow-up (16 men, 5 women), had baseline diagnosis of cancer, stroke, or coronary heart disease (4693 men, 6911 women), or pregnant at baseline interview (10 women). Very few women had ever smoked cigarettes (2113, 2.8%), and were excluded to avoid confounding by cigarette use. To reduce possible influence of reverse causation on our study results, we excluded participants with less than two years of follow-up after the baseline survey (590 men, 471 women) or BMI<18.5 (2607 men, 2571 women). After these exclusions (not mutually exclusive) data from 53425 Shanghai Men’s Health Study participants and 63017 Shanghai Women’s Health Study participants were available for analysis.

Sex-specific analyses were conducted in consideration of major differences between men and women in the distribution of anthropometric measurements, smoking, and drinking habits. Frequencies and distributions of the participant characteristics were tabulated for the total sample and by waist-hip ratio quintiles. Hazard ratios and 95% confidence intervals were estimated using Cox proportional hazard models for the association between central adiposity measures and mortality using age as the time scale with entry time defined as age at baseline interview and exit time as age at death or December 31, 2011 [21]. Waist-hip ratio was categorized into quintiles, with the smallest waist-hip ratio group serving as the reference group in analyses. We evaluated the proportional hazards assumption graphically and found no evidence of apparent departure. Statistical models were stratified by birth year (categorized into five-year groups) and adjusted for the following potential confounders identified *a priori*: education (≤ elementary, middle school, high school, > high school, unknown), occupation (professionals/technicians/administrators, clerical/service workers, manufacturing/agricultural workers), BMI (18.5 to 19.9, 20.0 to 22.4, 22.5 to 24.9, 25.0 to 27.4, ≥ 27.5), regular exercise (inactive or active at the sex-specific activity median (women: <1.2 metabolic equivalent (MET) hours/day, ≥1.2 MET-hours/day; men: <2.0 MET-hours/day, ≥2.0 MET-hours/day)), fruit and vegetable intake (sex-specific tertiles for women: <415.0 g/day, 415.0 to 638.4 g/day, ≥638.5 g/day; for men: <363.1 g/day, 363.1 to 557.3 g/day, ≥557.4 g/day), saturated fat intake (sex-specific tertiles for women: <6.45g/day, 6.5 to 9.7 g/day, ≥9.8 g/day; for men: <7.9 g/day, 7.9 to 11.3 g/day, ≥11.4 g/day), alcohol consumption (women: never, ever—due to the small number of women (N = 1399) who ever drank; men: never, former, current), height (sex-specific quintiles for women: ≤ 153 cm, 154 to 155 cm, 155 to 159 cm, 160 to 162 cm, ≥ 163 cm; for men: ≤ 165 cm, 166 to 169 cm, 170 to 171 cm, 172 to 175 cm, ≥ 176 cm), menopausal status (women only) and smoking status (men only: never, former, current < 20 cigarettes/day, current ≥ 20 cigarettes/day). Postmenopausal status was defined as not having a menstrual period for ≥ 12 months before baseline. Statistical models to test for trends in risk of death included an ordinal term to represent central adiposity groupings. We used restricted cubic spline regression to evaluate the shape of the association of waist-hip ratio and BMI with all-cause mortality [22], with four knots, placed at the 5^th^, 35^th^, 65^th^, and 95^th^ percentiles of the anthropometric measurement sex-specific distributions.

To further elucidate the relationship between waist-hip ratio and mortality, we stratified analyses by factors hypothesized to modify the association such as age at baseline, BMI, physical activity, smoking status, comorbidity, and duration of follow-up. We evaluated potential effect modification on the association between central adiposity and mortality by using likelihood ratio tests to compare main effects models with and without the addition of a cross-product interaction term. Statistical analyses were performed using SAS statistical software (version 9.3; SAS Institute Inc, Cary, NC).

## Results

There were 2058 deaths in the Shanghai Men’s Health Study after a median follow-up time of 7.5 years (range: 2 to 10 years) and 3167 deaths in the Shanghai Women’s Health Study with a median follow-up time of 13.2 years (range: 2 to 15 years). The most common causes of death over the study period were CVD (561 men, 802 women) and cancer (1031 men, 1569 women). The majority of the cohort was of normal BMI (median baseline BMI of 23.8 for men and 23.7 for women) and did not participate in regular exercise (66.0% of men and 66.4% of women). Many men in the cohort were current smokers (60.5%). On average, male participants with smaller waist-hip ratios were more likely to have lower BMI and an occupation in manufacturing or agricultural work than males with larger waist-hip ratios (Table 1). Female participants with smaller waist-hip ratios were younger, had lower BMI, higher education, and were less likely to be regular exercisers or to have an occupation in manufacturing or agricultural work than those with larger waist-hip ratios. BMI was highly correlated with waist circumference (Spearman r men = 0.81; women = 0.82) and moderately with waist-hip ratio (Spearman r men = 0.52; women = 0.45).

**Table 1:** Baseline Characteristics by Quintiles of Waist-hip Ratio, Shanghai, China, Shanghai Men’s Health Study 2002–2006, Shanghai Women’s Health Study 1996–2000.

		Waist-hip ratio quintiles				
	Men	<0.86	0.86–0.89	0.90–0.91	0.92–0.94	≥0.95
Characteristic (%)	(N = 53425)	(N = 10770)	(N = 10710)	(N = 10575)	(N = 10765)	(N = 10605)
Age (median, IQR, years)	52.2 (14.0)	51.4 (13.5)	52.0 (13.5)	52.0 (13.3)	52.2 (13.5)	53.7 (15.4)
BMI (median, IQR, kg/m^2^)	23.8 (3.8)	21.5 (3.0)	23.1 (3.1)	23.9 (3.2)	24.5 (3.2)	25.9 (3.5)
Smoking status						
Never	30.0	31.0	31.4	30.6	29.5	27.5
Former	9.5	7.4	9.7	9.1	9.9	11.6
Current <20 cigarettes/day	30.0	32.8	30.5	30.3	29.9	26.4
Current ≥20 cigarettes/day	30.5	28.8	28.4	30.0	30.7	34.5
Ever alcohol drinker	33.9	30.6	32.6	33.7	35.5	37.1
Daily intake (median, IQR, g)						
Saturated fat	9.6 (5.7)	9.5 (5.7)	9.6 (5.6)	9.7 (5.6)	9.6 (5.6)	9.6 (5.9)
Fruit and vegetables	456.6 (310.4)	441.0 (310.9)	456.6 (308.6)	461.1 (308.3)	466.2 (306.6)	455.4 (314.4)
Regular exerciser	34.0	33.7	35.0	34.0	34.0	33.1
Education						
≤Elementary school	5.6	5.6	5.6	4.9	5.2	6.9
Middle school	33.2	33.7	31.8	32.8	33.3	34.3
High school	36.4	36.0	36.6	37.0	37.5	34.9
> High school	23.4	22.7	24.4	24.2	22.9	22.8
Occupation						
Professionals, Technicians, Administrators	25.9	24.7	26.2	25.7	26.0	26.9
Clerical, Service	22.0	21.3	21.6	22.1	22.4	22.6
Manufacturing, Agricultural	52.1	54.1	52.2	52.2	51.7	50.5
	Women	<0.78	0.78–0.79	0.80–0.82	0.83–0.85	≥0.86
	(N = 63017)	(N = 12664)	(N = 12282)	(N = 12844)	(N = 12 801)	(N = 12 426)
Age (median, IQR, years)	49.2 (14.5)	46.1 (9.3)	47.4 (10.9)	48.9 (13.2)	50.8 (15.6)	55.8 (16.6)
BMI (median, IQR, kg/m^2^)	23.7 (4.2)	21.8 (3.3)	22.8 (3.4)	23.7 (3.7)	24.6 (3.9)	25.9 (4.2)
Ever alcohol drinker	2.0	1.7	1.8	2.1	2.2	2.2
Daily intake (median, IQR, g)						
Saturated fat	8.1 (5.1)	8.6 (5.1)	8.4 (5.0)	8.1 (5.0)	7.9 (5.2)	7.4 (5.1)
Fruit and vegetables	521.9 (355.8)	538.7 (353.1)	530.3 (347.9)	526.3 (351.8)	520.2 (358.9)	492.3 (364.3)
Regular exerciser	33.6	31.7	31.6	33.1	33.8	37.8
Education						
≤Elementary school	18.6	8.5	11.7	16.0	21.7	35.4
Middle school	38.8	39.9	40.5	39.7	39.1	34.7
High school	28.8	34.9	32.2	30.0	26.3	20.7
> High school	13.7	16.7	15.6	14.3	12.9	9.2
Occupation						
Professionals, Technicians, Administrators	28.6	32.4	30.9	29.8	27.5	22.4
Clerical, Service	20.9	21.0	21.0	21.1	20.6	20.9
Manufacturing, Agricultural	50.4	46.6	48.1	49.0	51.8	56.7

IQR = interquartile range.

**Fig 1:**
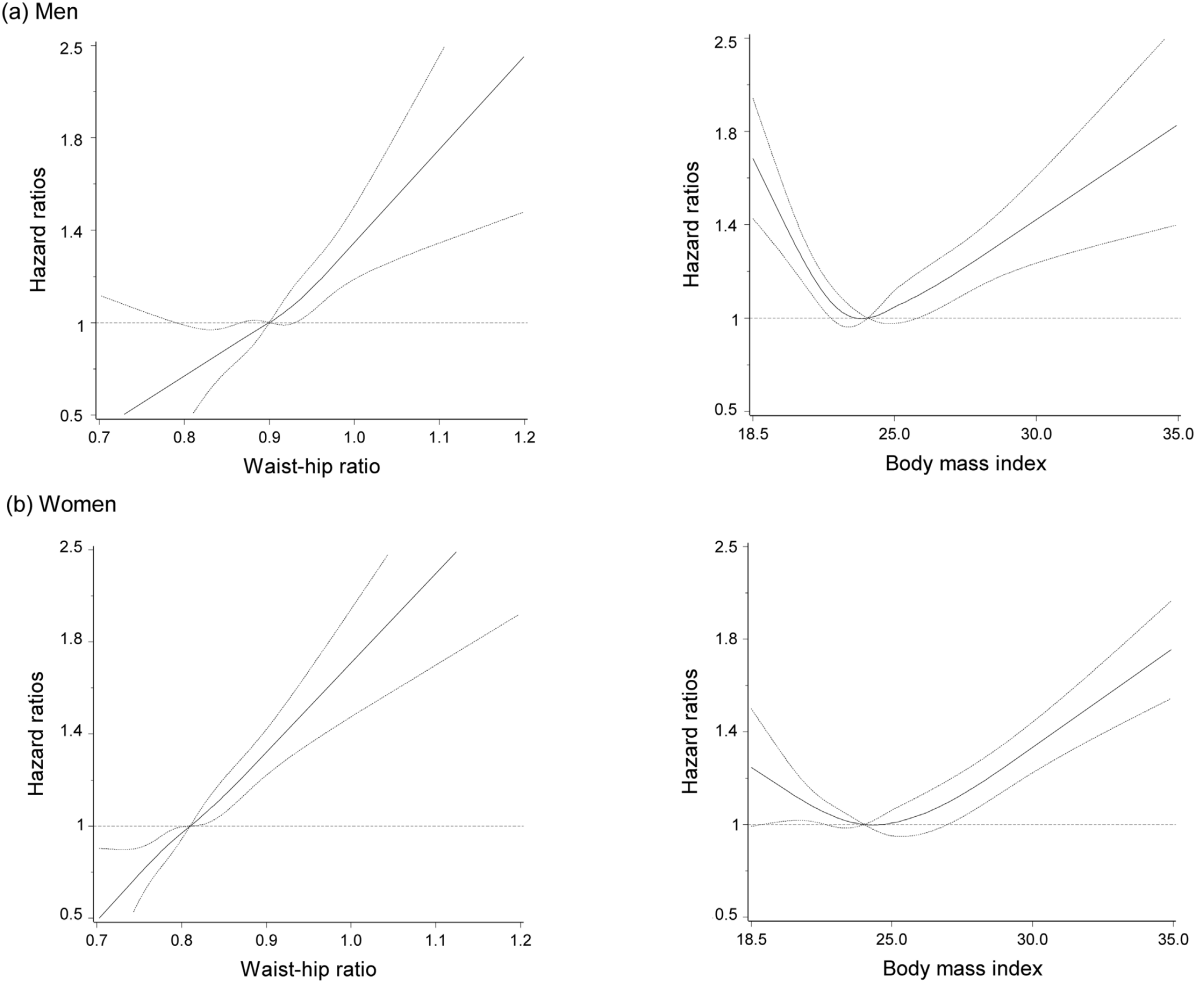
Multivariate hazard ratios for all-cause mortality in (a) men and (b) women by waist-hip ratio and body mass index, Shanghai, China, Shanghai Men’s Health Study 2002–2006, Shanghai Women’s Health Study 1996–2000. X-axes display adiposity measures; Y-axes display hazard ratio risk values for all-cause mortality plotted using the logarithmic scale. Body mass index hazard ratios are adjusted for education, occupation, regular exercise, dietary intake of saturated fats, fruits and vegetables, alcohol consumption, height, menopausal status (women only), and smoking status (men only). Waist-hip ratios are additionally adjusted for body mass index. Sex-specific medians were used as reference points: waist-hip ratio = 0.90 for men and 0.81 for women; body mass index = 23.8 for men, and 23.7 for women.

In age-adjusted models waist-hip ratio was modestly associated with all-cause mortality (Table 2). Adjustment for potential confounders except BMI did not materially change point estimates. After adjustment for BMI, a clear dose-response relationship between waist-hip ratio and all-cause mortality was observed (Fig 1 and Table 2). For both men and women when BMI was modeled continuously, in comparisons to median BMI the middle of the distribution showed a modest increased all-cause mortality risk and there was more notable increased mortality at both ends of the BMI distribution (Fig 1). The fully-adjusted HRs across the first to fifth quintiles of waist-hip ratio for men were 1 (reference), 1.10, 1.21, 1.11, and 1.42 and for women 1 (reference), 1.10, 1.11, 1.20, and 1.48 (*P-trends* < 0.001). Similarly, increasing waist circumference was associated with increasing all-cause mortality risk (data not shown).

**Table 2:** Risk of All-cause Mortality According to Waist-hip Ratio Quintiles, Shanghai, China, Shanghai Men’s Health Study 2002–2006, Shanghai Women’s Health Study 1996–2000.

	No. of subjects	No. of deaths	HR ^a^	95%CI	*P*_Trend_	HR ^b^	95%CI	*P*_Trend_	HR ^c^	(95%CI)	*P*_Trend_
Men	(N = 53425)	(N = 2058)									
< 0.86	10770	391	1	Referent		1	Referent		1	Referent	
0.86–0.89	10710	378	0.98	0.85, 1.13		1.00	0.87, 1.16		1.10	0.95, 1.27	
0.90–0.91	10575	386	1.04	0.91, 1.20		1.08	0.94, 1.24		1.21	1.04, 1.41	
0.92–0.94	10765	373	0.96	0.84, 1.11		0.98	0.85, 1.13		1.11	0.96, 1.30	
≥0.95	10605	530	1.24	1.09, 1.42	0.003	1.24	1.09, 1.41	0.004	1.42	1.22, 1.65	<0.001
Women	(N = 63017)	(N = 3167)									
<0.78	12664	366	1	Referent		1	Referent		1	Referent	
0.78–0.79	12282	442	1.09	0.95, 1.25		1.07	0.93, 1.23		1.10	0.96, 1.27	
0.80–0.82	12844	545	1.10	0.96, 1.25		1.07	0.93, 1.22		1.11	0.97, 1.27	
0.83–0.85	12801	687	1.19	1.05, 1.35		1.14	1.00, 1.30		1.20	1.05, 1.37	
≥0.86	12426	1127	1.53	1.36, 1.73	<0.001	1.43	1.27, 1.62	<0.001	1.48	1.30, 1.69	<0.001

^a^ HRs are estimated using Cox models with age as the time scale and stratified by birth year.

^b^ HRs are further adjusted for education, occupation, regular exercise, dietary intake of saturated fats, fruits and vegetables, alcohol consumption, height, menopausal status (women only), and smoking status (men only).

^c^ HRs are additionally adjusted for body mass index.

In men and women, positive associations were observed between waist-hip ratio and the cause-specific mortality outcomes examined: cardiovascular diseases, cancer, and other-causes; although the association observed between waist-hip ratio and cancer mortality was relatively weak (Table 3). Chronic obstructive pulmonary disease (93 men and 61 women) and diabetes (70 men and 183 women) were the most common causes of deaths in the other-causes of death group.

**Table 3:** Risk of Cause-specific Mortality According to Waist-hip Ratio Quintiles, Shanghai, China, Shanghai Men’s Health Study 2002–2006, Shanghai Women’s Health Study 1996–2000.

	CVD mortality						Cancer mortality						Other mortality ^a^					
	No. of deaths	HR ^b^	95%CI	HR ^c^	95%CI	*P*_Trend_	No. of deaths	HR ^b^	95%CI	HR ^c^	95%CI	*P*_Trend_	No. of deaths	HR ^b^	95%CI	HR ^c^	95%CI	*P*_Trend_
Men	(N = 561)						(N = 1,031)						(N = 466)					
<0.86	88	1	Referent	1	Referent		203	1	Referent	1	Referent		100	1	Referent	1	Referent	
0.86–0.89	99	1.18	0.88, 1.57	1.18	0.88, 1.58		197	1.01	0.83, 1.22	1.12	0.91, 1.37		82	0.85	0.63, 1.13	0.98	0.73,1.33	
0.90–0.91	113	1.43	1.08, 1.89	1.41	1.05, 1.90		189	1.00	0.82, 1.22	1.15	0.94, 1.42		84	0.92	0.68, 1.22	1.14	0.84,1.55	
0.92–0.94	102	1.21	0.91, 1.61	1.18	0.87, 1.60		202	1.01	0.83, 1.22	1.17	0.95, 1.45		69	0.72	0.53, 0.97	0.93	0.67,1.29	
≥0.95	159	1.67	1.28, 2.16	1.54	1.14, 2.07	0.01	240	1.06	0.88, 1.28	1.25	1.01, 1.55	0.05	131	1.23	0.94, 1.59	1.72	1.27,2.32	0.002
Women	(N = 802)						(N = 1569)						(N = 796)					
<0.78	67	1	Referent	1	Referent		219	1	Referent	1	Referent		80	1	Referent	1	Referent	
0.78–0.79	91	1.14	0.83, 1.56	1.16	0.84, 1.59		232	0.97	0.80, 1.17	0.98	0.81, 1.18		119	1.31	0.98, 1.74	1.41	1.06,1.88	
0.80–0.82	136	1.29	0.96, 1.73	1.30	0.96, 1.75		287	1.00	0.83, 1.19	1.00	0.84, 1.20		122	1.08	0.81, 1.43	1.24	0.93,1.66	
0.83–0.85	175	1.33	1.00, 1.77	1.33	0.99, 1.78		339	1.04	0.87, 1.23	1.04	0.87, 1.24		173	1.27	0.97, 1.66	1.52	1.15,2.01	
≥0.86	333	1.80	1.37, 2.35	1.70	1.28, 2.26	<0.001	492	1.22	1.04, 1.44	1.20	1.01, 1.44	0.01	302	1.64	1.27, 2.11	2.06	1.57,2.69	<0.001

CVD = cardiovascular disease.

^a^ Other mortality included deaths from causes other than cardiovascular disease and cancer.

^b^ HRs are estimated using Cox models with age as the time scale, stratified by birth year and adjusted for education, occupation, regular exercise, dietary intake of saturated fats, fruits and vegetables, alcohol consumption, height, menopausal status women only, and smoking status men only.

^c^ HRs are additionally adjusted for body mass index.

**Fig 2:**
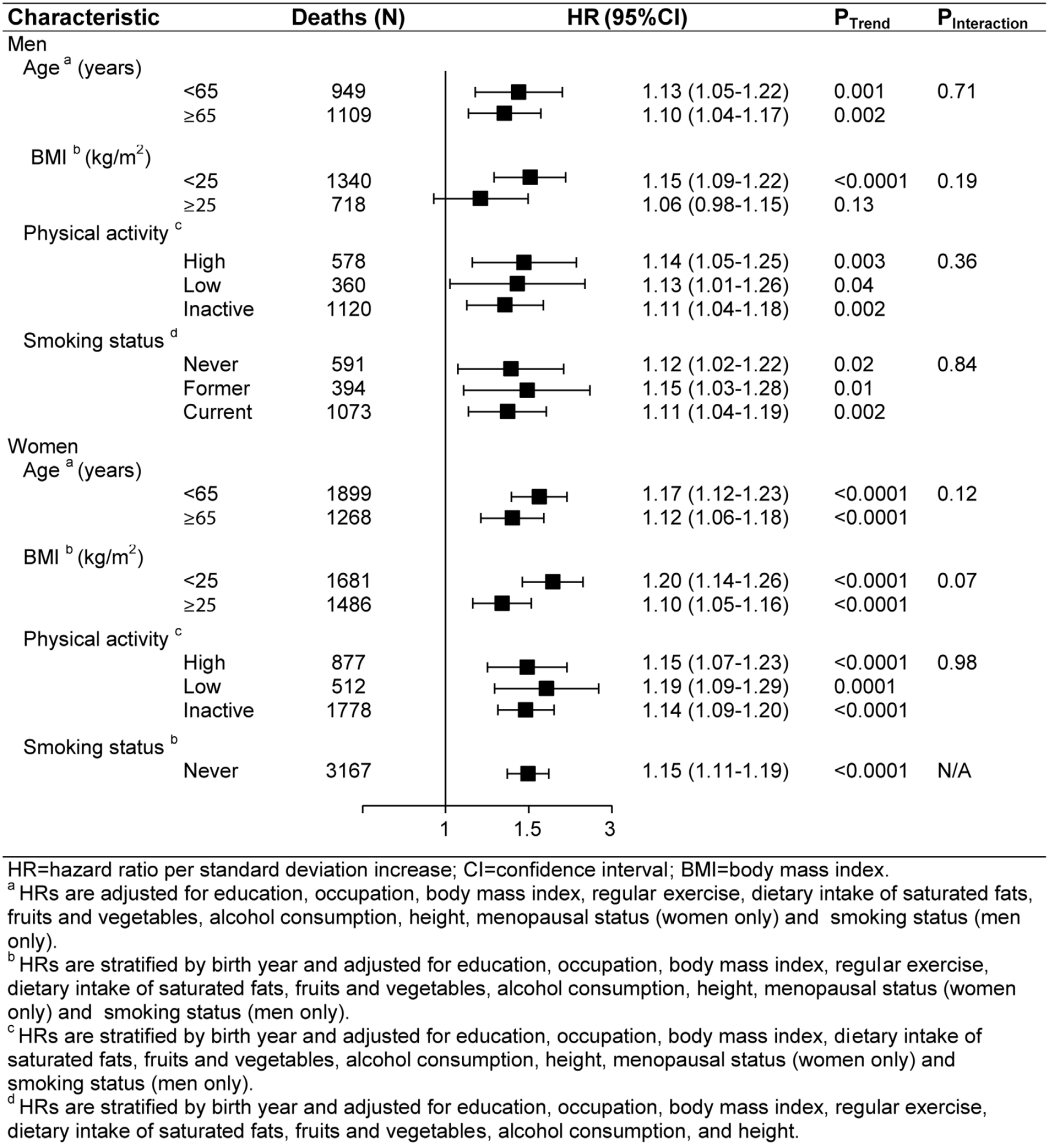
Risk of all-cause mortality according to waist-hip ratio stratified by selected factors, Shanghai, China, Shanghai Men’s Health Study 2002–2006, Shanghai Women’s Health Study 1996–2000. Hazard ratios for the association between all-cause mortality and waist-hip ratio stratified by age, body mass index, physical activity and smoking status. Point estimates are plotted on the logarithmic scale.

**Fig 3:**
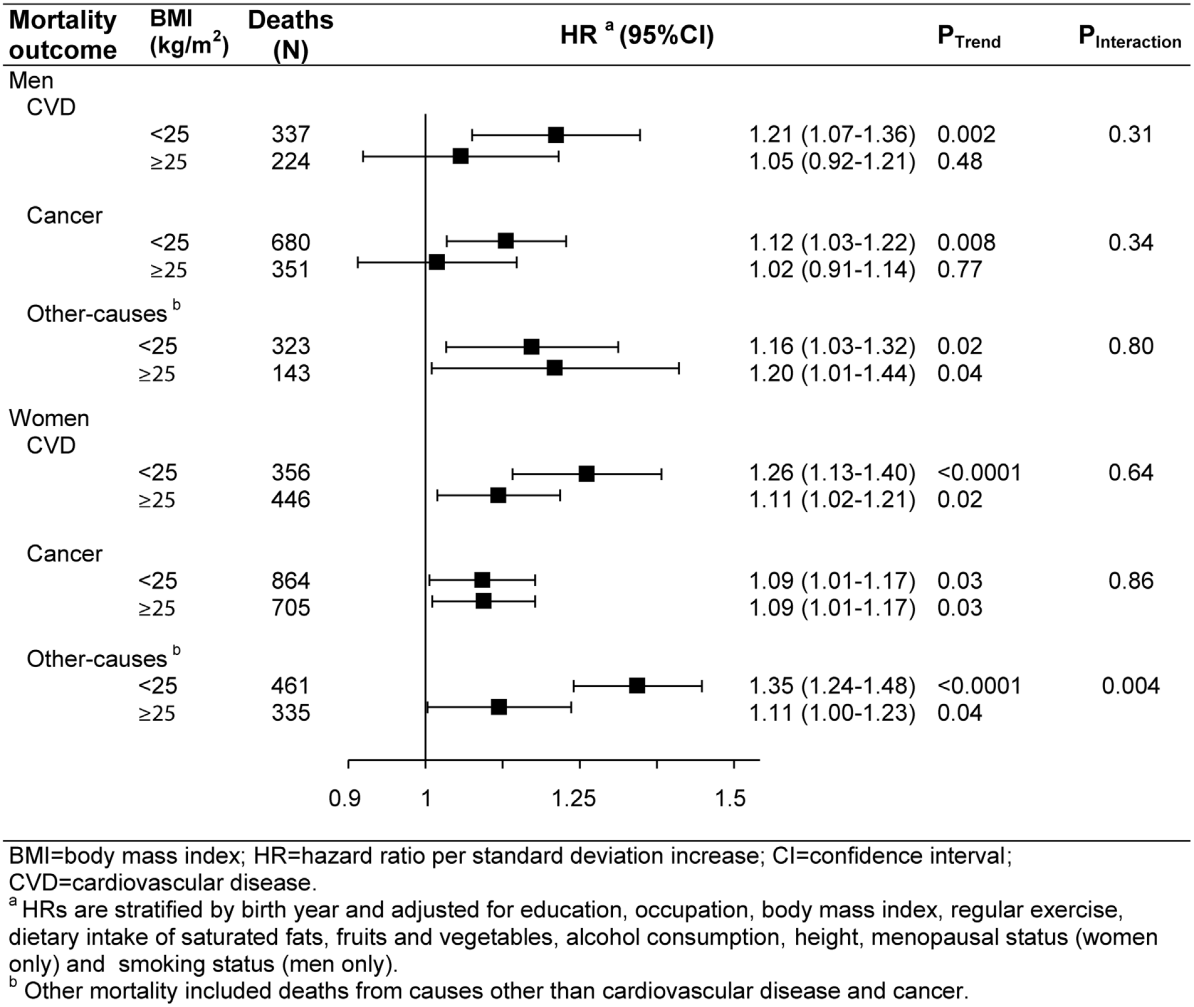
Risk of cause-specific mortality according to waist-hip ratio stratified by body mass index, Shanghai, China, Shanghai Men’s Health Study 2002–2006, Shanghai Women’s Health Study 1996–2000. Hazard ratios for the associations between waist-hip ratio with cardiovascular disease, cancer and other-cause mortality, stratified by baseline body mass index. Point estimates are plotted on the logarithmic scale.

We evaluated effect modification of the association between central adiposity and mortality by baseline BMI, age, physical activity, and smoking status. In BMI stratified analyses, robust associations were observed between waist-hip ratio and all-cause death, CVD death and other-cause death in normal weight participants (Figs 2 and 3). Weaker and null associations were observed in men with BMI ≥ 25.0 for all-cause, CVD, and cancer mortality. Correspondingly, women with BMI ≥ 25.0 experienced weaker, although significant, associations with all-cause, CVD, and other-causes mortality. Formal tests for multiplicative interactions between BMI and waist-hip ratio quintiles, however, were mostly statistically non-significant at *P-interaction* > 0.05, perhaps due to a small sample size among those with a high BMI but a low waist-hip ratio. No apparent effect modification of baseline age or physical activity were observed on the associations between central adiposity and all-cause mortality in either sex (Fig 2). Among men, we evaluated possible effect modification by smoking and did not find a significant multiplicative interaction (*P-interaction* = 0.84).

Sensitivity analyses were conducted to evaluate the strength of the association between waist-hip ratio and all-cause mortality in subgroups defined by chronic disease status. The associations between waist-hip ratio and all-cause mortality were consistent regardless of baseline existing diabetic or hypertensive conditions (Table 4). In women the association between waist-hip ratio and all-cause mortality was similar for deaths which occurred within five years and more than five years after study enrollment; in men the association was slightly stronger in deaths more than five years post enrollment (data not shown).

**Table 4:** Risk of All-cause Mortality According to Waist-hip Ratio Quintiles and Disease Status at Baseline, Shanghai, China, Shanghai Men’s Health Study 2002–2006, Shanghai Women’s Health Study 1996–2000.

	Diabetes or hypertension diagnosis at baseline				Without diabetes or hypertension diagnosis at baseline			
	No. of deaths	HR ^a^	95%CI	*P*_Trend_	No. of deaths	HR ^a^	95%CI	*P*_Trend_
Men	(N = 952)				(N = 1106)			
<0.86	129	1	Referent		262	1	Referent	
0.86–0.89	152	0.95	0.75, 1.21		226	1.14	0.95, 1.37	
0.90–0.91	170	1.05	0.82, 1.33		216	1.26	1.04, 1.52	
0.92–0.94	203	1.11	0.88, 1.41		170	0.99	0.80, 1.22	
≥0.95	298	1.19	0.94, 1.50	0.04	232	1.50	1.23, 1.85	0.005
Women								
	(N = 1302)				(N = 1865)			
<0.78	87	1	Referent		279	1	Referent	
0.78–0.79	144	1.12	0.85, 1.46		298	1.06	0.90, 1.25	
0.80–0.82	186	0.98	0.76, 1.27		359	1.13	0.96, 1.33	
0.83–0.85	289	1.10	0.86, 1.40		398	1.18	1.01, 1.39	
≥0.86	596	1.49	1.18, 1.89	<0.001	531	1.34	1.14, 1.57	<0.001

^a^ HRs are stratified by birth year and adjusted for education, occupation, body mass index, regular exercise, dietary intake of saturated fats, fruits and vegetables, alcohol consumption, height, menopausal status (women only) and smoking status (men only).

## Discussion

In this prospective study of Chinese adults, greater central adiposity is associated with increased risk of cardiovascular disease mortality, cancer mortality, and all-cause mortality in a dose-response manner. In both men and women a 1.5-fold elevated risk of all-cause death is found in comparisons between the largest and the smallest waist-hip ratio quintiles underscoring the detrimental association between large waist-hip ratio and death. This positive association is observed regardless of baseline age, comorbidity, or smoking status in men. There are several biologic mechanisms that may explain the association between excess central adiposity and higher mortality, including chronic inflammation and insulin resistance [23].

Compared with the results obtained from an early analysis of Shanghai Women’s Health Study data [20], the associations between central adiposity and mortality are somewhat attenuated after seven additional years of follow-up. For example, hazard ratios for the comparison of the fifth to first waist-hip ratio quintiles in the Shanghai Women’s Health Study are 1.95 (95%CI: 1.60, 2.38) in the earlier report compared to 1.48 (95%CI: 1.30, 1.69) in the current study. In contrast to the previous Shanghai Women’s Health Study project, we present results that exclude participants with baseline cancer, stroke, and coronary heart diseases as well as use slightly different confounder adjustments. Importantly, with the longer follow-up period, there have been more deaths causing point estimates and confidence intervals to be more precise. The present study is the first of its size to evaluate the relationship between central adiposity and mortality in Chinese men.

We present central adiposity and mortality associations both with and without adjustment for BMI. Our models suggest that when BMI is held constant participants with larger waist-hip ratio are at increased mortality risk. Mortality risk associated with high waist-hip ratio for a given BMI may reflect the consequences of increased abdominal/visceral fat. Alternatively, the increased risk may indicate low muscle tone and peripheral fat for a given BMI in those with a high waist-hip ratio. We are unable to distinguish between these two possibilities without further study and a more comprehensive measurement of body fat and composition. We also investigated potential interaction between BMI and waist-hip ratio. Although tests for interactions between waist-hip ratio and BMI were mostly non-significant, the association between central adiposity and all-cause mortality is most apparent in normal weight BMI participants. The European Prospective Investigation into Cancer and Nutrition (EPIC) cohort study found a slightly stronger association between central adiposity measures and mortality in participants with lower BMI values [2]. Both the EPIC results and our findings highlight the importance of using central adiposity for risk assessment among individuals with normal BMI.

Similar to previous studies, we did not observe effect modification of the association between waist-hip ratio and mortality by physical activity [24], or smoking status (among men) [2,15]. One previous study reported a stronger association between waist circumference and all-cause mortality among less physically-active men; however, the authors were unable to evaluate the association with waist-hip ratio [15]. Former and current male smokers in the EPIC study [2] had a somewhat stronger association between waist circumference and all-cause mortality (*P-interaction* = 0.02), however, smoking did not modify the positive association between waist-hip ratio and all-cause mortality in EPIC men or women (*P-interaction* = 0.79 in men and 0.57 in women) [2].

Epidemiologic studies of adiposity and mortality are susceptible to reverse causation. Study participants with the lowest BMI are usually a heterogeneous group of individuals including smokers and chronically ill participants [25]. To address the potential for reverse causation we exclude participants with BMI less than 18.5, who died within two years of the baseline interview, or who had a diagnosis of cancer, stroke, or heart disease at baseline. Additionally, in this study the strength of the association between all-cause mortality and waist-hip ratio is similar by age and comorbidity status at baseline, and for the total follow-up period, the first five years, and greater than five years of cohort follow-up. The consistent association in sensitivity analyses suggests that reverse causality is not driving the relationship between central adiposity and mortality in this study. Furthermore, similar associations are observed in the men’s cohort where prevalence of smoking is high and in the women’s cohort that includes only non-smokers, suggesting that the true underlying relationship between waist-hip ratio and mortality is consistent across sex and smoking patterns. Although the response rate at baseline recruitment for the men’s cohort study was lower than the women’s cohort study, the 74% response rate achieved for the men’s cohort study is higher than most existing large cohort studies. Ensuring high internal validity is one of the top priorities of any cohort study. The internal validity of our study results should be high given the very high follow-up rate of both cohorts. A low response rate at the baseline recruitment may affect the ability to generalize the finding to the general population (external validity). However, given the high response rate achieved at the baseline recruitment for both cohort studies, we have no reason to speculate that the finding from this study cannot be generalized to the general population. The follow-up time for the women’s cohort is longer than the men’s cohort. However, the strength of the association between WHR and mortality outcomes was similar in these two cohorts. With an extended follow-up of the men’s cohort, we should be able to evaluate the longer-term association between WHR and mortality outcomes in the men’s cohort.

## Conclusions

Results from our large prospective population-based studies find a linear association between central adiposity and CVD and all-cause mortality. The association is more evident for participants with a normal weight BMI than those with an overweight BMI at baseline. Central adiposity is a major public health concern and an important predictor of mortality risk in Chinese adults, as it is in North American and European adults. Our data emphasize the need to go beyond BMI and to incorporate measures of central fat distribution in assessing the heath impact of the rising obesity epidemic.

## References

[1] Haslam DW, James WPT. Obesity. Lancet. 2005;366: 1197–1209. 10.1016/S0140-6736(05)67483-1 16198769

[2] Pischon T, Boeing H, Hoffmann K, Bergmann M, Schulze MB, Overvad K, et al General and abdominal adiposity and risk of death in Europe. N Engl J Med. 2008;359: 2105–2120. 10.1056/NEJMoa0801891 19005195

[3] Prospective Studies Collaboration, Whitlock G, Lewington S, Sherliker P, Clarke R, Emberson J, et al Body-mass index and cause-specific mortality in 900 000 adults: collaborative analyses of 57 prospective studies. Lancet. 2009;373: 1083–1096. 10.1016/S0140-6736(09)60318-4 19299006 PMC2662372

[4] Adams KF, Leitzmann MF, Ballard-Barbash R, Albanes D, Harris TB, Hollenbeck A, et al Body mass and weight change in adults in relation to mortality risk. Am J Epidemiol. 2014;179: 135–144. 10.1093/aje/kwt254 24173550 PMC3873112

[5] Berrington de Gonzalez A, Hartge P, Cerhan JR, Flint AJ, Hannan L, MacInnis RJ, et al Body-mass index and mortality among 1.46 million white adults. N Engl J Med. 2010;363: 2211–2219. 10.1056/NEJMoa1000367 21121834 PMC3066051

[6] Zheng W, McLerran DF, Rolland B, Zhang X, Inoue M, Matsuo K, et al Association between body-mass index and risk of death in more than 1 million Asians. N Engl J Med. 2011;364: 719–729. 10.1056/NEJMoa1010679 21345101 PMC4008249

[7] WHO Expert Consultation. Appropriate body-mass index for Asian populations and its implications for policy and intervention strategies. Lancet. 2004;363: 157–163. 10.1016/S0140-6736(03)15268-3 14726171

[8] Bei-Fan Z, Cooperative Meta-Analysis Group of Working Group on Obesity in China. Predictive values of body mass index and waist circumference for risk factors of certain related diseases in Chinese adults: study on optimal cut-off points of body mass index and waist circumference in Chinese adults. Asia Pac J Clin Nutr. 2002;11 Suppl 8: S685–693.

[9] Ntuk UE, Gill JMR, Mackay DF, Sattar N, Pell JP. Ethnic-specific obesity cutoffs for diabetes risk: cross-sectional study of 490,288 UK biobank participants. Diabetes Care. 2014;37: 2500–2507. 10.2337/dc13-2966 24974975

[10] Lim U, Ernst T, Buchthal SD, Latch M, Albright CL, Wilkens LR, et al Asian women have greater abdominal and visceral adiposity than Caucasian women with similar body mass index. Nutr Diabetes. 2011;1: e6 10.1038/nutd.2011.2 23449381 PMC3302135

[11] Wang J, Thornton JC, Russell M, Burastero S, Heymsfield S, Pierson RN Jr. Asians have lower body mass index (BMI) but higher percent body fat than do whites: comparisons of anthropometric measurements. Am J Clin Nutr. 1994;60: 23–28. 8017333 10.1093/ajcn/60.1.23

[12] Folsom AR, Kaye SA, Sellers TA, Hong CP, Cerhan JR, Potter JD, et al Body fat distribution and 5-year risk of death in older women. JAMA J Am Med Assoc. 1993;269: 483–487. 8419667

[13] Simpson JA, MacInnis RJ, Peeters A, Hopper JL, Giles GG, English DR. A comparison of adiposity measures as predictors of all-cause mortality: the Melbourne Collaborative Cohort Study. Obes Silver Spring Md. 2007;15: 994–1003. 10.1038/oby.2007.622 17426335

[14] Zhang C, Rexrode KM, van Dam RM, Li TY, Hu FB. Abdominal obesity and the risk of all-cause, cardiovascular, and cancer mortality: sixteen years of follow-up in US women. Circulation. 2008;117: 1658–1667. 10.1161/CIRCULATIONAHA.107.739714 18362231

[15] Jacobs EJ, Newton CC, Wang Y, Patel AV, McCullough ML, Campbell PT, et al Waist circumference and all-cause mortality in a large US cohort. Arch Intern Med. 2010;170: 1293–1301. 10.1001/archinternmed.2010.201 20696950

[16] Reis JP, Macera CA, Araneta MR, Lindsay SP, Marshall SJ, Wingard DL. Comparison of overall obesity and body fat distribution in predicting risk of mortality. Obes Silver Spring Md. 2009;17: 1232–1239. 10.1038/oby.2008.664 19197258

[17] Cerhan JR, Moore SC, Jacobs EJ, Kitahara CM, Rosenberg PS, Adami H-O, et al A pooled analysis of waist circumference and mortality in 650,000 adults. Mayo Clin Proc. 2014;89: 335–345. 10.1016/j.mayocp.2013.11.011 24582192 PMC4104704

[18] Cai H, Zheng W, Xiang Y-B, Xu WH, Yang G, Li H, et al Dietary patterns and their correlates among middle-aged and elderly Chinese men: a report from the Shanghai Men’s Health Study. Br J Nutr. 2007;98: 1006–1013. 10.1017/S0007114507750900 17524168

[19] Zheng W, Chow W-H, Yang G, Jin F, Rothman N, Blair A, et al The Shanghai Women’s Health Study: rationale, study design, and baseline characteristics. Am J Epidemiol. 2005;162: 1123–1131. 10.1093/aje/kwi322 16236996

[20] Zhang X, Shu X-O, Yang G, Li H, Cai H, Gao Y-T, et al Abdominal adiposity and mortality in Chinese women. Arch Intern Med. 2007;167: 886–892. 10.1001/archinte.167.9.886 17502529

[21] Korn EL, Graubard BI, Midthune D. Time-to-event analysis of longitudinal follow-up of a survey: choice of the time-scale. Am J Epidemiol. 1997;145: 72–80. 8982025 10.1093/oxfordjournals.aje.a009034

[22] Desquilbet L, Mariotti F. Dose-response analyses using restricted cubic spline functions in public health research. Stat Med. 2010;29: 1037–1057. 10.1002/sim.3841 20087875

[23] Després J-P, Lemieux I. Abdominal obesity and metabolic syndrome. Nature. 2006;444: 881–887. 10.1038/nature05488 17167477

[24] Bellocco R, Jia C, Ye W, Lagerros YT. Effects of physical activity, body mass index, waist-to-hip ratio and waist circumference on total mortality risk in the Swedish National March Cohort. Eur J Epidemiol. 2010;25: 777–788. 10.1007/s10654-010-9497-6 20730597

[25] Willett WC, Hu FB, Colditz GA, Manson JE. Underweight, overweight, obesity, and excess deaths. JAMA J Am Med Assoc. 2005;294: 551; author reply 552–553. 10.1001/jama.294.5.551-a 16077044

